# Abatacept as salvage therapy in chronic graft-versus-host disease—a retrospective analysis

**DOI:** 10.1007/s00277-021-04434-x

**Published:** 2021-01-30

**Authors:** Tobias Wertheimer, Marius Dohse, Gabriel Afram, Daniela Weber, Martin Heidenreich, Barbara Holler, Anna-Sophia Kattner, Andreas Neubauer, Stephan Mielke, Per Ljungman, Ernst Holler, Wolfgang Herr, Matthias Edinger, Antonio Pérez Martínez, Matthias Fante, Daniel Wolff

**Affiliations:** 1 Department of Internal Medicine III, Hematology and Oncology, University Hospital Regensburg, Franz-Josef-Strauss-Allee 11, 93053 Regensburg, Germany; 2 Department of Hematology, Oncology and Immunology, University Hospital Giessen and Marburg, Marburg, Germany; 3 Department of Cellular Therapy and Allogeneic Stem Cell Transplantation, Karolinska University Hospital and Karolinska Institutet, Stockholm, Sweden; 4 Regensburg Center for Interventional Immunology, Regensburg, Germany; 5 Pediatric Hematology, Oncology and Stem Cell Transplantation, Hospital Universitario La Paz, Universidad Autonóma de Madrid, Madrid, Spain

**Keywords:** Chronic graft-versus-host disease, Abatacept, Salvage therapy, Bronchiolitis obliterans syndrome

## Abstract

The immunomodulatory fusion protein abatacept has recently been investigated for the treatment of steroid-refractory chronic graft-versus-host disease (cGvHD) in a phase 1 clinical trial. We analyzed the safety and efficacy of abatacept for cGvHD therapy in a retrospective study with 15 patients who underwent allogeneic hematopoietic stem cell transplantation (allo-HSCT) and received abatacept for cGvHD with a median age of 49 years. Grading was performed as part of the clinical routine according to the National Institute of Health’s (NIH) consensus criteria at initiation of abatacept and 1, 3, 6, 9 and 12 months thereafter. The median time of follow-up was 191 days (range 55–393 days). Best overall response rate (ORR) was 40%. In particular, patients with bronchiolitis obliterans syndrome showed significant clinical improvement and durable responses following abatacept treatment with a response rate of 89% based on improvement in lung severity score (*n* = 6) or stabilized lung function (*n* = 4) or both (*n* = 3). Infectious complications CTCAE °III or higher were observed in 3/15 patients. None of the patients relapsed from the underlying malignancy. Thus, abatacept appears to be a promising treatment option for cGvHD, in particular for patients with lung involvement. However, further evaluation within a phase 2 clinical trial is required.

## Introduction

While allogeneic hematopoietic stem cell transplantation (allo-HSCT) is a well-established, potentially curative therapy for several malignant and benign hematologic diseases, chronic graft-versus-host disease (cGvHD) remains a major complication after allo-HSCT. cGvHD occurs in up to 70% of patients after allo-HSCT and significantly contributes to impaired quality of life and non-relapse mortality (NRM)/transplant-related mortality (TRM) [1–5].

Corticosteroids represent the backbone of cGVHD treatment, but contribute to an already high morbidity and mortality by causing complications such as osteoporosis, myopathy, avascular necrosis, and glaucoma [3, 6]. A substantial number of patients does not respond to corticosteroids alone and require second-line therapy with the recently FDA-approved Bruton’s tyrosine kinase inhibitor ibrutinib or extracorporeal photopheresis [5, 7–9]. Further treatment options are usually based on retrospective or phase 1/2 clinical studies and show limited efficacy in a significant proportion of patients despite harboring the risk of toxicity including infectious complications [10–14].

Abatacept is a novel, first in class immunomodulatory drug exerting its effect by costimulatory blockade and is applied for the treatment of rheumatoid arthritis and other rheumatological diseases [15–17]. It is a recombinant fusion protein comprised of the extracellular domain of the immune checkpoint protein cytotoxic T lymphocyte-associated protein 4 (CTLA-4) fused to the Fc fragment of IgG_1_ [18]. By binding with high affinity to the costimulatory receptors CD80 and CD86 on antigen presenting cells (APCs), it counteracts the costimulatory signal mediated by the ligand CD28, which is required for full T cell activation [19].

The highly complex pathophysiology of cGvHD is still poorly understood. Apart from alloreactive donor T cell responses, it involves aberrant innate immune signaling, endothelial cell injury, dysfunctional central tolerance induction (due to thymic damage as a result of the conditioning regimen or alloreactive T cells), insufficient de novo development of regulatory T cells (T_reg_), dysregulation of B cells, and cytokine signaling eventually resulting in chronic inflammation and fibrotic remodeling [20–22].

Since cGvHD is at least in part mediated by host reactive T cells stimulated by allogeneic antigens [23], there is high rationale for abatacept as a treatment option in cGvHD, and it has been reported that CTLA-4 blockade can prevent aGvHD and cGvHD and even reverse cGvHD in murine models [24]. Recently, abatacept has received breakthrough approval by the American Food and Drug Administration (FDA) for the prevention of acute GvHD and has shown efficacy in a phase 1 clinical trial for patients with steroid-refractory cGvHD, albeit with relatively low patient numbers [25].

Therefore, we analyzed the efficacy and safety of abatacept for the treatment of advanced cGvHD in a multicentric retrospective study.

## Patients and methods

### Patients

In this retrospective analysis, patients treated with abatacept for cGvHD between 2018 and 2020 at the University Hospital Regensburg (Germany), University Hospital Giessen and Marburg (Germany), University Hospital and Karolinska Institutet (Stockholm, Sweden) and University Hospital La Paz (Madrid, Spain) were included into the analysis approved by the institutional ethics review board (no.19-1586-104). Documentation of cGvHD was performed as part of clinical routine using the diagnosis and response criteria according to the National Institute of Health (NIH) consensus guidelines [26]. No new immunosuppressive agent was applied within at least 4 weeks before abatacept therapy, and response assessment was discontinued upon requirement of any additional immunosuppressive treatment post abatacept therapy. Patients received abatacept intravenously at a dose of 10 mg/kg body weight (maximum 800 mg) every 2 weeks for the first three doses and then every 4 weeks.

### Definition of abatacept response and adverse events

Response to abatacept was assessed at 1, 3, 6, 9, and 12 months after start of therapy. In case of treatment with an additional immunosuppressive agent, the last response assessment for abatacept was performed at onset of new therapy. Last follow-up of the present analysis was January 2020. Complete remission (CR) was defined as resolution of all organ manifestations of cGvHD. Improvement of at least one organ grade without progression of cGvHD at other organs was classified as partial response (PR); mixed response was defined as simultaneous improvement in one organ and progression in another organ. Patients who showed no change in organ grading were classified as stable disease (SD). Failure-free survival (FFS) was defined as absence of relapse or non-relapse mortality or addition of further systemic therapy. Overall response rates (ORR) were calculated based on intention to treat analysis. Infectious complications were assessed and classified according to the common terminology criteria for adverse events version 5.0 (CTCAE 5.0) with toxicities captured in the analysis starting from grade III.

## Results

### Patient characteristics

We treated 15 patients with abatacept for cGvHD at our centers between 2018 and 2020. Among those patients, the diagnoses leading to allo-HSCT were myeloid disorders (acute myeloid leukemia (AML), myelodysplastic syndrome (MDS), myeloproliferative neoplasia (MPN)) in 10 patients, lymphatic malignancies (acute lymphoblastic leukemia (ALL), and Hodgkin lymphoma (HL)) in four patients and one patient suffering from CTLA-4 haploinsufficiency.

All patients had received peripheral blood stem cells (PBSC) as graft source with 11 patients grafted from an HLA-matched sibling (5 patients) or an unrelated donor (8 patients). HLA-matched donors were defined as 10/10 match. One patient had received an HLA-C mismatched graft from an unrelated donor, and one patient received a haploidentical graft from a related donor. Acute GvHD grade II or higher according to Glucksberg criteria occurred in 11 patients (85%). cGvHD onset was quiescent in 8 patients, de novo in three patients and progressive in four patients. Most of the patients (*n* = 12; 80%), who received abatacept, had severe cGvHD. One patient included in the analysis had mild cGvHD (prior history of moderate cGvHD) and received abatacept due to intolerance to other immunosuppressive agents. One patient was treated for autoimmune-hemolytic anemia (AIHA) refractory to corticosteroids and rituximab not fulfilling the NIH criteria for cGvHD falling in the category “undefined other cGvHD” [26, 27]. The majority of the patients had steroid dependent cGvHD (*n* = 10, 67%, all others steroid-refractory cGvHD (*n* = 5; 33%)). A platelet count < 100/nl was observed in 4 patients (27%) at the time of abatacept initiation.

The most common organ manifestations of cGvHD were skin (*n* = 11; 73%), lung (*n* = 11; 73%), eyes (*n* = 10; 67%), and oral cavity (*n* = 10; 67%). Abatacept was initiated on median day 1848 (range 432–7953) after allo-HSCT and on day 1592 (range 28–7864) after cGvHD onset, respectively. The corticosteroid dose at abatacept initiation was 0.34 mg/kg in median (range 0,12–2 mg/kg). The patients included in the analysis had received a median of 4.6 prior treatment lines (range 2–5) for cGvHD. Within 3 months before abatacept treatment was started, most patients did not undergo new immunosuppressive treatments (80%). The remaining patients (20%), who underwent new immunosuppressive treatments within 3 months, were progressive or refractory to the initiated treatment.

Median follow-up after treatment was 179 days (range 55–393). Patient characteristics including age, gender, diagnosis, donor type, stem cell source, conditioning regimen, GvHD prophylaxis, history of acute GvHD, and chronic GvHD are shown in Table 1.

**Table 1:** Patient characteristics

Patient characteristics	Value
Patients	15
Female, *n* (%)	10
Male, *n* (%)	5
Age, median (range)*	49 (5–70)
Diagnosis	
AML, *n* (%)	7 (47)
ALL, *n* (%)	2 (13)
MPN (%)	3 (15)
Others, *n* (%)	3 (15)
Donor type	
HLA-matched unrelated, *n* (%)	7 (47)
HLA-mismatched unrelated, *n* (%)	1 (7)
HLA-matched related, *n* (%)	6 (40)
HLA-mismatched related, *n* (%)	0
Haploidentical related, *n* (%)	1 (7)
Sex mismatch: female to male	
Yes, *n* (%)	1 (7)
No, *n* (%)	14 (93)
Stem cell source	
Peripheral blood stem cells, *n* (%)	14 (93)
Bone marrow, *n* (%)	1 (7)
GvHD prophylaxis	
ATG/CsA/MTX, *n* (%)	3 (20)
CsA/MTX, *n* (%)	5 (33)
CsA/MMF, *n* (%)	1 (7)
Other	6 (40)
History of aGvHD	
Grades 0–I, *n* (%)	4 (27)
Grades II–IV, *n* (%)	11 (73)
Characteristics	Value
Onset of cGvHD after allo-SCT, median days (range)	256 (76–597)
Time point of abatacept treatment after allo-SCT, median days, (range)	1848 (432–7953)
Time point of abatacept treatment after cGvHD onset, median days, (range)	1592 (133–7864)
Age at abatacept initiation, median (range)	49 (5–70)
Number of abatacept doses, median (range)	7.1 (1–20)
cGvHD, *n* (%)	
Mild	1 (7)
Moderate	0
Severe non-NIH defined (AIHA)	13 (87) 1 (7)
Steroid response of cGvHD	
Steroid resistance	5 (33)
Steroid dependence	10 (67)
Number of organ involvement of cGvHD, *n* (%)	
One	1 (7)
Two	1 (7)
Three	5 (33)
Four or more	8 (53)
Type of cGvHD organ involvement, *n* (%)	
Skin	11 (73)
Oral	10 (67)
Eyes	10 (67)
Liver	2 (13)
Gut	4 (27)
Lung	11 (73)
Musculoskeletal	7 (47)
Genital	2 (13)
AIHA	1 (7)
ISM at the beginning of abatacept, *n* (%)	
No ISM	0
One ISM	5 (33)
Two ISM	8 (53)
Three or more ISM	2 (13)
Number of prior therapies before abatacept, *n* (%)	
One	2 (13)
Two	2 13)
Three	1 (7)
Four or more prior therapies	10 (67)
New ISM within 3 months before abatacept, *n* (%)	
Yes	3 (20)
No	12 (80)

*n* number of patients, *AML* acute myeloid leukemia, *ALL* acute lymphoblastic leukemia, *MPN* myeloproliferative neoplasia, *HLA* human leukocyte antigen, *ATG* anti-thymocyte globulin, *CsA* ciclosporin A, *MTX* methotrexate, *MMF* mycophenolate mofetil, *GvHD* graft-versus-host disease, *aGvHD* acute GvHD, *cGvHD* chronic GvHD, *ISM* immunosuppressive medication

### Response to abatacept

**Fig. 1:**
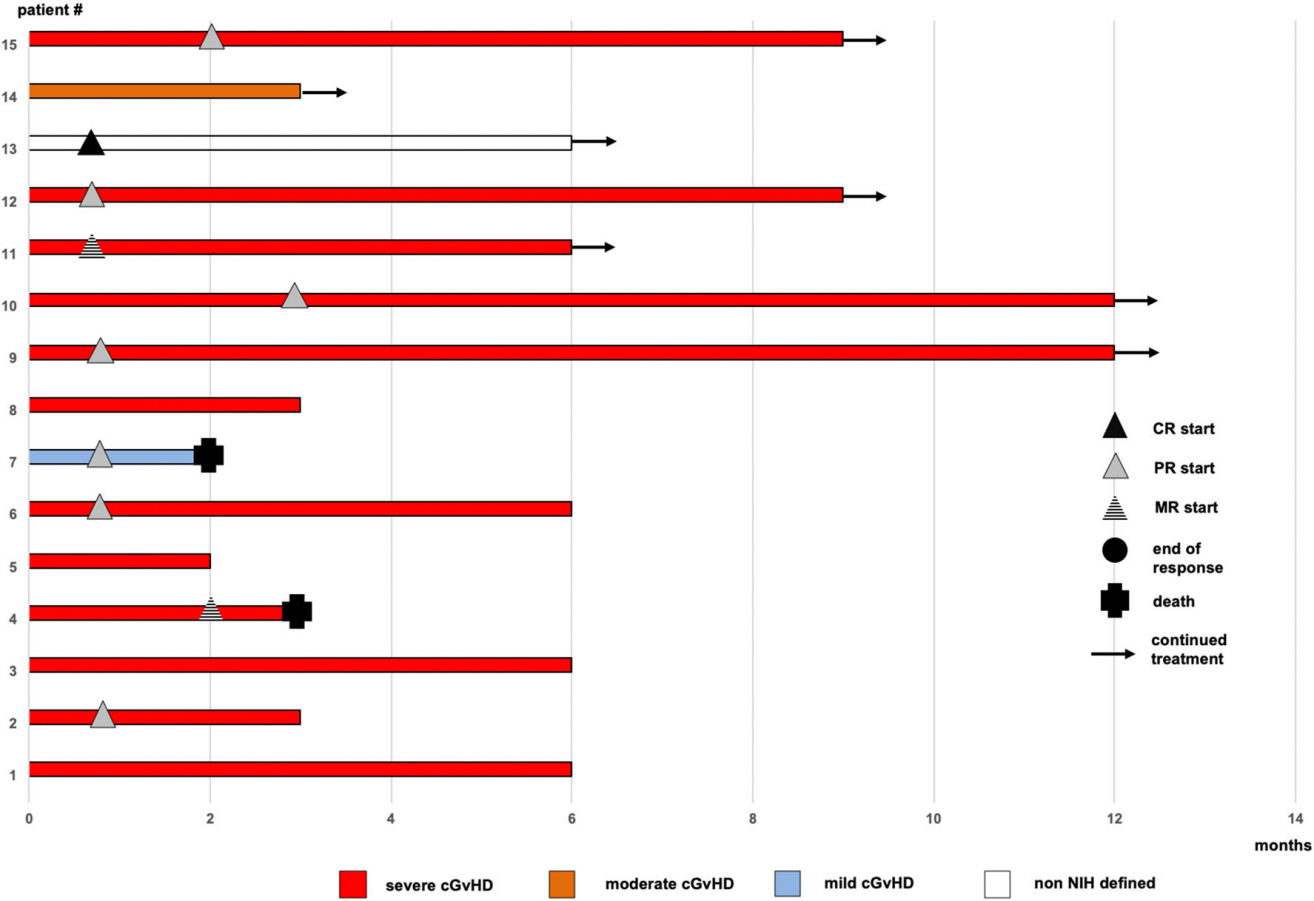
Clinical course during abatacept treatment

Response to abatacept treatment is illustrated in Fig. 1 and Table 2 at the different time points of this analysis.

**Table 2:** Duration of abatacept treatment and clinical course

Patient #	Day of start	IS at start	# of doses	Organ involvement (grade) at start	Skin features at start	1 m RR	2 m RR	3 m RR	6 m RR	9 m RR	12 m RR	Follow up
1	7953	GC, E, MMF	7	s (0), e (3), m (0), f (0), lu (3)	-	SD	SD	SD	SD	-	-	Stabilized lung function, abatacept discontinued due to prolonged fatigue and diarrhea after administration
2	1596	GC, Rux	6	s (3), e (1), m (1), f (2), lu (2)	S	PR	PR	PR	**-**	-	-	Improvement of lung GvHD, new IS with ibrutinib and tocilizumab after 6 months
3	3261	GC, CsA	2	s (3), e (0), m (2), f (2), lu (0)	S	SD	SD	SD	SD	-	-	New IS with daratumumab after no improvement of oral ulcers
4	1425	GC	4	s (3), e (1), m (1), f (2), lu (2)	S	SD	MR	SD	-	-	-	Death due to aortic valve rupture (preexisting aortic valve insufficiency)
5	949	GC, Rux	3	s (3), e (2), m (0), f (2), lu (0)	S	SD	SD	-	-	-	-	New IS with Treg after 2 months
6	722	GC	10	s (0), e (3), m (0), f (0), lu (3)	-	PR	PR	PR	PR	-	-	Stabilized lung GvHD and improved functional capacity, death due to sepsis 9 months after abatacept start
7	1318	GC	1	s (0), e (0), m (1), f (0), lu (0)	-	PR	PR	-	-	-	-	Abatacept was given due to intolerance to IS agents and multiple infectious complications; death due to infectious complications of PAD
8	4587	GC, Bari	5	s (3), e (0), m (0), f (2), lu (0)	S	SD	SD	SD	-	-	-	New IS with tofacitinib after persisting arthralgia
9*	1311	GC, Ima	14	s (1), e (1), m (1), gi (1), f (0), lu (3)	L	PR	PR	PR	PR	PR	PR	Durable improvement of lung and skin GvHD. Less oxygen demand, reduced coughing
10*	432	GC, E	14	s (3), e (1), m (1), f (0), lu (0)	S	SD	SD	PR	PR	PR	PR	Improvement of ocular GvHD
11	1162	GC, Tac	9	s (1), e (3), m (1), f (0), lu (3)	L	MR	MR	MR	MR	-	-	Stabilized lung function, progressive oral affection, new IS with ruxolitinib after 9 months
12*	517	Toc	13	s (2), m (1), gi (1), li (1), f (0), lu (3)	L	PR	PR	PR	PR	PR	PR	Complete resolution of s, m, gi and li GvHD. Significant improvement of lung GvHD (reduction from grades 3 to 1)
13*	157	GC	4	AIHA	-	CR	CR	CR	CR	NR	NR	Complete resolution of AIHA
14*	469	GC, Ibru		s (0), e (0), m (1), gi (0), f (0), lu (1)	-	SD	SD	SD	NR	NR	NR	Stabilized lung function
15*	1864	CsA	10	s (1), e (1), m (2), gi (0), f (2), lu (3)	L	SD	PR	PR	PR	PR	NR	Increased mobility and improved lung symptoms
Median	1848		7									

*1/3/6/9/12-month RR* 1/3/6/12-month response rate, respectively; *GC* glucocorticoid; *E* everolimus; *MMF* mycophenolate mofetil; *Rux* ruxolitinib; *CsA* cyclosporine A; *Bari* baricitinib; *Ima* imatinib; *Tac* tacrolimus; *Toc* tocilizumab; *Ibru* ibrutinib; *s* skin; *e* eyes; *m* mouth; *f* fascia; *lu* lungs; *ge* genital; *S* sclerotic features; *L* lichen planus-like features; *PD* progressive disease; *SD* stable disease; *PR* partial response; *MR* mixed response; *NR* not reached; *Treg* regulatory T cells; *IS* immunosuppression; *PAD* peripheral artery disease; *QOL* quality of life; *AIHA* autoimmune hemolytic anemia; *ongoing therapy

### Response to abatacept at 1 month

One month after first administration of abatacept, the patient (7%) with AIHA showed CR, 5 (33%) patients had PR, one patient (7%) showed MR with improved lung function but progressive oral affection, and 8 patients (53%) presented with SD. None of the patients required start of an additional immunosuppressive therapy. The overall response rate (ORR) at 1 month was 40%, and failure-free survival (FFS) was 100%.

### Response to abatacept at 3 months

At 3 months after start of abatacept therapy, one patient showed CR (7%), 5 (33%) patients had PR, one patient showed MR (7%), and 6 patients (40%) had SD. One patient who had SD started a new immunosuppression and another patient with a PR succumbed to infectious complications of severe peripheral artery disease unrelated to abatacept. ORR at 3 months was therefore 40% with an FFS of 87%.

### Response to abatacept at 6 and 9 months

The 6 months follow-up was reached by nine patients with one patient remaining in CR (7%), four patients achieving PR (27%), one MR (7%) with sustained stabilized lung function but impaired oral affection, and two patients with SD (13%). At termination of our analysis, one patient was still treated with abatacept but has not reached the 6 months follow-up yet. One patient with PR started a new IS with ibrutinib and tocilizumab, and another patient who was in SD discontinued abatacept and was switched to tofacitinib due to persisting arthralgia. One highly comorbid patient with preexisting aortic valve insufficiency died due to aortic valve rupture. Thus, the ORR at 6 months was 33% with an FFS of 64%.

Nine months after initiation of abatacept treatment, four patients still received therapy and two patients currently still receiving abatacept have not reached the time point yet. Of the remaining patients, three showed a PR (20%), one patient discontinued abatacept due to sustained and not improved oral ulcers, and another patient died due to urosepsis, displaying an ORR of 23% with an FFS of 31%.

### Response to abatacept at 12 months

At 12 months, three patients were still treated with abatacept, and three who currently receive abatacept therapy have not reached the time point yet. Three patients still had PR (25%) resulting in an ORR of 25% with FFS of 25%.

### Response in patients with lung involvement of cGvHD

**Fig. 2:**
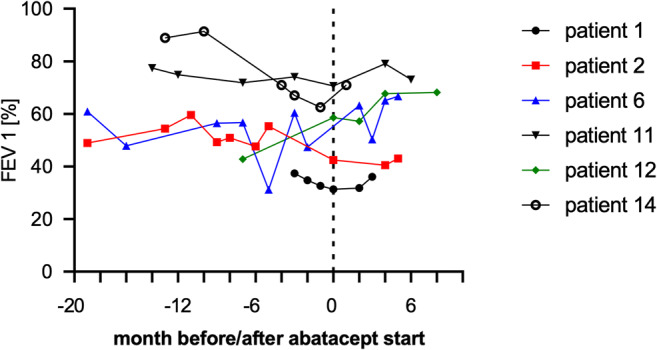
Forced expiratory volume % before and during abatacept therapy

Interestingly, we observed that patients with lung involvement (*n* = 9) particularly benefitted from therapy with abatacept with an overall response rate of 89% based on improvement in lung severity score (*n* = 6), lung function as measured by FEV1 in Fig. 1 (*n* = 4) or both (*n* = 3). Of note, although only a stabilization on abatacept was achieved, they experienced prior constant loss of FEV1 as shown in Fig. 1. Of note, all patients received in parallel therapy with FAM (fluticasone, azithromycine, montelukast) partly in combination with beta-agonists which had already been applied > 1 month before abatacept initiation without response (Fig. 2).

### Infectious and other complications during abatacept

Adverse events (AE) and serious adverse events (SAE) during abatacept treatment are illustrated in Table 3. In general, abatacept administration was well tolerated; however, one patient repeatedly showed nausea, vomiting, diarrhea, fever, and fatigue for several days repeatedly upon infusion leading to termination of treatment after 7 applications. Another patient showed alopecia during treatment. Three of the patients included in the analysis developed significant infectious complications requiring hospital admission with one highly comorbid patient succumbing to urosepsis, and another patient already mentioned died due to infectious complications associated with severe peripheral artery disease unrelated to cGvHD.

**Table 3:** Adverse and serious adverse events of abatacept treatment

Patient #	AE	SAE	Specification
1	Yes	No	Fatigue, diarrhea, fever
2	No	Yes	Death due to gram-negative sepsis 70 days after last abatacept dose
3	No	No	-
4	No	No	-
5	No	No	-
6	No	Yes	Death due to urosepsis 24 days after last abatacept dose
7	No	Yes	Death due to infectious complications of peripheral artery disease
8	No	No	-
9	No	No	-
10	Yes	No	Alopecia after abatacept initiation
11	No	No	-
12	No	Yes	Hospital admission due to influenza A pneumonia
13	No	No	-
14	No	No	-
15	Yes	No	Bone pain after infusion

## Discussion

cGvHD occurs with an incidence of 30–70% in patients undergoing allo-HSCT [21], and as it reduces quality of life and significantly contributes to NRM/TRM, there is still a high clinical need for effective second-line treatments [28, 29]. The underlying complex pathophysiology of cGvHD involves both B and T cell immunity and results in pleiotropic clinical manifestations resembling various autoimmune diseases [21]. Due to the involvement of auto- und alloreactive T cells in the development and course of cGvHD, there is high rationale for the use of costimulation blockade via the CTLA-4 pathway [20]. The introduction of the immunomodulatory drug abatacept has significantly improved the therapy for rheumatoid arthritis patients not responding sufficiently to conventional disease modifying antirheumatic drugs and has in this context shown efficacy and improvement of quality of life [19, 30, 31]. Based on observations in preclinical models, abatacept has been tested in combination with a CD25 monoclonal antibody in pediatric recipients of haploidentical allo-HSCT for the treatment of hyperacute GvHD, where it has shown efficacy [32]. Moreover, the safety and efficacy of abatacept for the prevention of aGvHD and treatment of SR-cGvHD were recently evaluated with promising results in phase 1 clinical trials [25, 33], and subsequent randomized phase 2 trials have been initiated (NCT0174313; NCT01954979).

In this retrospective analysis of cGvHD patients treated with abatacept at four centers, we observed a best overall response rate of 40%, which is comparable to the clinical response rate of 44% recently reported by Nahas et al. [25]. Despite the low number of patients and the retrospective character of the analysis, we observed that in particular patients with bronchiolitis obliterans syndrome (BOS) showed substantial clinical improvement after abatacept application with durable responses, stabilized lung function (Fig. 1), albeit this was not reflected by an improvement in lung grading in all patients. Given the frequently irreversible character of lung involvement due to fibrotic remodeling, we consider clinical improvement, lowering of oxygen demand, and stabilized lung function as relevant response parameters, especially in our patient cohort with mostly severe cGVHD [34]. Interestingly, it has been reported for several autoimmune diseases that costimulation blockade of the CTLA-4 axis selectively decreases the proportion of T follicular helper cells, thereby reducing T cell help for germinal center B cells [35–37]. Given that BOS is explicitly characterized by disturbance of B cell homeostasis with increased CD19^+^CD21^-^ B cells and excess of B cell activation factor (BAFF) [38], abatacept might target Tfh cells in this context, which would at least partially explain the particular improvement of patients with BOS in our study. In addition, we observed a complete response in a steroid and rituximab refractory AIHA patient as previously described [39]. Despite not meeting NIH diagnostic criteria for cGvHD, we included this patient in our analysis since it has recently been reported that based on biomarker profiles, patients with signs of immune mediated damage not diagnostic for cGvHD do not significantly differ from those showing diagnostic signs of cGvHD suggesting that the current NIH diagnostic criteria may not involve all targets of cGvHD [40, 41]. Noteworthy, in the phase 1 clinical trial reported by Nahas et al., a reduction of corticosteroid usage of 51.3% was reported, while the authors state that this effect might have been overestimated due to the not blinded or randomized design of the study. In this regard, we did not observe consistent steroid reduction in patients responding to abatacept, yet most of our patients received a relatively low corticosteroid dose at start of abatacept. Interestingly, it has been reported in a preclinical model of chronic lung allograft dysfunction that bronchiolitis obliterans can be attenuated by CTLA-4-Ig administration presumably by promoting LAG3^+^Treg mediated anti-inflammatory effects providing a potential mechanistic explanation for the observed clinical response [42]. Of note, progression of cGvHD within three months was indicative for treatment failure, and all patients, who achieved at least a PR, were responding in the first three months. Thus, based on our experience, we would suggest discontinuing abatacept treatment, if patients do not show a response within this period. Overall, abatacept seems to be a relevant treatment option for patients with cGvHD, particularly for patients with BOS, but this has to be further investigated in future clinical trials.
